# The Effect of a Voice-Centered Psycho-Educational Program on Maternal Self-Efficacy: A Feasibility Study

**DOI:** 10.3390/ijerph18052537

**Published:** 2021-03-04

**Authors:** Helen Shoemark, Marie Dahlstrøm, Oscar Bedford, Lauren Stewart

**Affiliations:** 1 Boyer College of Music and Dance, Temple University, Philadelphia, PA 19122, USA; 2 Center for Music in the Brain, Aarhus University, 8000 Aalborg, Denmark; mdahl001@clin.au.dk; 3 Montreal Neurological Institute, McGill University, Montreal, QC H3A 0G4, Canada; oscar.bedford@mail.mcgill.ca; 4 Department of Psychology, Goldsmiths University of London, London SE14 6NW, UK; l.stewart@gold.ac.uk

**Keywords:** maternal self-efficacy, contingent singing, music, community-based intervention, feasibility trial

## Abstract

This study examined the effect of a brief psycho-educational program, *Time Together*, on maternal self-efficacy, mother-infant bonding, and mood/anxiety for community-based mothers. This program centered on maternal voice, timing of interplay, and recognition of infant cues. A convergent parallel mixed-methods design included quantitative measures: the Karitane Parenting Confidence Scale, the Mother-Infant Bonding Scale, Edinburgh Postnatal Depression Scale and State & Trait Anxiety Inventory, and a sequential qualitative analysis to elaborate on the quantitative findings. Significant changes on the Karitane Parenting Confidence Scale were found. Qualitative analysis of the participant interviews and reflective diaries from the two weeks following the psycho-educational program confirmed that participation enhanced mothers’ ability to understand their infant, to soothe their infant when distressed, to play and to establish an effective bedtime routine. This feasibility study indicated that this is a promising approach to improve early mother-infant interaction and maternal self-efficacy.

## 1. Introduction

Building on Bandura’s personal efficacy theory [1,2], parental self-efficacy (PSE) is thought to be a key cognitive construct in parenting, reflecting the beliefs parents hold about their capabilities of successfully executing parenting tasks [3,4]. As such, self-efficacy increases when parents experience success in completing a task, and decreases after experiencing failure [5]. Parental self-efficacy can strongly influence parenting behavior [6] such that parents with high self-efficacy exhibit warmer and less over reactive behaviors towards their children [7,8] while those with low self-efficacy tend to lack the responsiveness required for contingent interaction [9,10].

Self-efficacy has been a target for parental psycho-educational programs, which seek to modify parental behaviors at the beginning of a parent-infant relationship. Such programs may be administered individually but are more typically group-based interventions which use didactic teaching, group discussions, demonstrations, role-play, and modelling of different aspects of infant care and parenting. Meta-analyses demonstrated that PSE is significantly enhanced by universal preventive psycho-educational programs, concluding that “providing universal parent education interventions that focus on enhancing PSE may be protective against the detrimental effects of fatigue and psychological distress that parents may experience” [11].

Psycho-educational programs have the potential to be most successful when they concentrate on a parent’s pre-existing capabilities and bring them to the fore. One specific capability that is universally present, and highly salient for the newborn, is a parent’s use of voice. It is well known that infants show strong preference for infant-directed speech, which is characterized by its musical features [12]. When caregivers talk to infants, they often modify their speaking voice, by widening their pitch range, altering timbre and adjusting content such as repeating appealing words to attract infant attention [13]. These alterations in the adult voice, are thought to convey positive affect [14] and elicit increased responsiveness from the infant [14,15], critical for infant neurodevelopment and language learning [16]. These features have been strategically applied to promote contingency in parent-infant interactions and infant neurodevelopment—often in explicitly therapeutic contexts [17]. When this is implemented as an intentional treatment protocol, it is described as Contingent Singing [18]. In Contingent Singing, specific musical attributes such as melody, register, dynamics, tempo, timbre, attack and silence are consciously manipulated by the music therapist to provide a balance between stimulation and support, contingent on the needs of the infant [17,18]. Moreover, the infant’s vocal, facial and gestural communications must be appropriately interpreted by the adult in order to maintain reciprocity in the interaction. Thus, Contingent Singing is the explicit construction of interplay in which the music therapist purposefully emulates the infant’s expressive capabilities of gesture, posture facial expression and evolving vocal capacity, to validate and encourage the infant’s earliest interactive capabilities. The positive effects of Contingent Singing have been assessed in the Neonatal Intensive Care Unit (NICU) setting by Malloch et al., who demonstrated that a music therapist implementing this method could facilitate the neuropsychological development of hospitalized newborn infants, as compared to a control group receiving standard care [19].

Contingent Singing has been translated into a parent psycho-educational protocol called ‘*Time Together*’ [20]. In *Time Together* the therapist validates and expands the existing expressive and receptive communicative capacities of new parents. It is designed to help mothers reflect upon infant and caregiver behaviors through a discussion of how the voice can create, support and modulate interplay with their infant. In particular, the content of the *Time Together* program includes consideration for the role that the maternal voice can play in mutual regulation within the mother-infant dyad, and encourages mothers to become aware of their infant’s cues for interaction in order to create and maintain an optimal range of interaction with their infant. Moreover, vocal interplay and singing provide a context in which mothers become aware of their own capabilities. In a qualitative feasibility trial of the *Time Together* program [20], mothers in a NICU reported feeling more equipped to interpret their infant’s cues and use their own voice to influence their infant’s state or interaction following the program. 

Although *Time Together* was initially developed for clinical contexts, primarily families in the NICU, the utility of this program for families in the community has yet to be explored. The development of services to support those at risk, or with less dramatic needs may serve as a preventative strategy to sustain or build capacity in parents. Specifically, new mothers often report depressive symptoms [21], anxiety [22] and feelings of low parental self-efficacy [10] in the perinatal period, all of which are potentially detrimental for child-rearing. The present study therefore investigated whether *Time Together* could influence parental self-efficacy, infant-bonding and parental mood/anxiety.

## 2. Materials and Methods

### 2.1. Design and participants 

The present study implemented a convergent parallel mixed-methods design, collecting quantitative data from twenty-two mothers between 25–43 years of age (*M* = 34.8, *SD* = 4.3) and their infants between 2 and 14 weeks of age (*M* = 9.6, *SD* = 2.5), both before and after participation in *Time Together*. The participants were recruited across two periods of one week each through Millfields Children’s Centre and a parent-infant event in East London (see Table 1). Parents were given a flyer and self-selected into the study by contacting the research team. The study received ethical approval from Goldsmiths Dept of Psychology Research Ethics Committee (PS220216MDS). 

### 2.2. Materials

Four validated self-report questionnaires were used. The Karitane Parenting Confidence Scale [23] measured perceived parent self-efficacy as the primary outcome. The Mother-Infant Bonding Scale (MIBS) [24] was used to assess the participants’ feelings towards their baby. The Edinburgh Postnatal Depression Scale (EPDS) [25] and State & Trait Anxiety Inventory (STAI) [26] were used to consider the anxiety and depression status of participants. The data analysis was undertaken sequentially, to allow the quantitative analysis to guide the deductive content analysis [27]. In vivo coding was used to explore participant’s reports to identify what they valued and experienced in the *Time Together* program. 

### 2.3. Procedure 

The *Time Together* program, lasting 90 min, was administered to groups of participants comprising 3–5 mother-infant dyads. The practitioner did not delve into a therapeutic process with the parent but promoted self-efficacy by gently inquiring about what the parent has noticed, experienced, or actually done, and used the parent’s existing capacity as the starting point for discussion about what else they could do. The information was designed to (a) help parents to interpret their infant’s gestural, postural, facial and vocal behaviors as demonstrations of the infant’s availability for interplay, and (b) promote awareness of how to use their expressive capacities in response to their infant’s behaviors, and (c) promote the parent’s awareness and a value for their capacity to respond to their infant’s cues in supportive and interactive ways. The expected outcome was the promotion of parental self-efficacy in dyadic interaction The program was administered by a qualified music therapist who was suitably skilled in the *Time Together* program which required skillful support, tailored information about parental and infant expressive behaviors, acceptance for the current status of what each participant knew, understood and could do (see Shoemark, 2018 for detailed description and sample). 

Participants completed the four questionnaires at two time points: immediately before and then two weeks after the *Time Together* program, when they also participated in a semi-structured follow-up interview. Each participant was also provided with a daily diary in which to reflect upon how they were using knowledge gained from the program in their everyday interactions with their infants. 

## 3. Results

Eighteen mothers and infants had a mean age of 34.8 years and 9.6 weeks respectively. The majority of mothers had completed undergraduate education and were first-time parents (89%), and the majority were currently employed (61%). Four participants were unable to complete the post-program questionnaires and were therefore excluded from the quantitative analysis.

Overall the assumptions of internal consistency were not violated in the majority of administered scales, besides the MIBS which displayed low internal consistency (α = 0.45, post-program). A Shapiro–Wilks test indicated a normal distribution of the data for the STAI and KPCS, and a non-normal distribution for the EPDS and MIBS, warranting the use of non-parametric statistics for these tests. Full baseline data of descriptive statistics are provided in Table 1. 

### 3.1. Quantitative Outcomes

Data was analyzed using Excel’s ‘Real Statistics’ package at group level. One-tailed *t*-tests were performed on the normally distributed scales, while one-tailed Wilcoxon signed rank tests were performed on the scales for which assumptions of normality were not met. Table 2 shows pre and post scores for the four measures of interest: EPDS, STAI, MIBS, KPCS. Results were significant for the KPCS (Z(18) = −1.078), *p* = <0.004). 

### 3.2. Qualitative Findings

As there was a significant effect for the KPCS (see Appendix A), the KPCS items were used as the categories for the deductive content analysis to give greater insight into participants’ experiences. Within these categories, participants’ comments from transcribed interviews and their daily journals were organized in themes. The participants’ comments were concentrated within the KPCS categories 2–7, and 13. 

Seventeen participants indicated new capacity under KPCS 5: “I understand what my baby is trying to tell me”. Within this category, the themes were as follows: 

(a) a clearer understanding of the experience for their baby—Eight participants specified they understood when “it’s too much” for their baby. Five participants suggested they were “more in tune with” their baby. Four participants noted they understood their baby more, with Participant 11 simply stating, “We understand each other better.” 

(b) The notion of timing was reported. Two participants specified they now know their baby’s early cues of distress, and four noted that they now realize their baby takes time to react to something new. Participant 8 realized that, “If I did something, and he didn’t do anything, I thought “that’s ok then, he’s fine with it”, but I realize now it’s more like he was still taking it in, he wasn’t sure yet” and at the other threshold when it’s “too much”, Participant 22 commented that it was valuable, “knowing when she is turning away, actually she is tired and she doesn’t need stimulation.” A distinction that “I understand him/her differently now” was made by a four participants, and Participant 12 reported, “My eyes are more open for what I’m doing, I’m noting more things at the moment, I can tell that I understand my baby a little bit more.” 

Further responses were grouped around the participants’ capacity to soothe and respond to their infant’s distress (KPCS 2: I can soothe my baby; KPCS 4: I know what to do when my baby cries; KPCS 6: I can soothe my baby when she is distressed). Participant 6 commented, “I am now able to diffuse most crying bouts by launching into song!”. Eight participants reported they had been singing songs to successfully soothe their baby, but also using other vocalizations such as the small chant (“sh-sh-sh”), and consciously using the reassuring high-to-low intonation. Participant 15 noted that “sort of really going from high to low when she was distressed seemed to really calm her down… so that was really good”. She and her partner had begun to sing at stressful times such as getting the baby to sleep or at bath time, thereby creating a reliable strategy. Participants used the diaries to report songs they had tested and confirmed as successful. Four participants reported effective use of chant (using their voice to chant little repeated sounds), a specific item from the program. Participant 6 reported she was now able to use her voice in a manner provided by the program to diffuse a previously stressful situation:

“There was always trigger moments, that he gets upset, like putting on his snowsuit. He got upset every single time and we’d leave the house in tears, and the neighbors would be looking out the windows at the woman with her baby crying again, and it always seemed like the baby was constantly crying. So I started singing to him when I put him in the snowsuit and that diffused it. It’s diffusing isn’t it… It kind of diffused the moment, and he’d start smiling so we’d leave the house not in tears, which was good.” 

A further cluster of responses were coded under KPCS 3, “I am confident about helping my baby to establish a good sleep routine”. Participant 20 explained, “In bedtime I now use my voice to over-ride her cries and to let her know that it is ok to relax while using a similar mantra and remaining calm and soothing”. Six participants explained that singing had quickly become part of a new ritual for putting their babies to sleep. Participant 12 said, “Straight away after the session I started singing to him, and now this is a part of my new routine. I use this to soothe him to sleep, and we have a ritual now, after you feed, I sing for him and soothe him, and he falls asleep because I sing.” Participant 16 revealed, “I feel I have gotten better at putting her to bed, calmly and gently and with singing, so that she stays calm and happy and soothes herself to sleep.”

For KPCS 7 (I am confident about playing with my baby), participants reported they began to take the time to notice what their baby enjoys, and commented that their babies “love” their singing and talking. They describe their play-time as having “real conversations”, and Participant 8 smiled, “He already seems to be reacting well to this—smiling every time I sing a new part of a song he’s heard a few times.”

The final cluster of responses was for KPCS 13 “I feel I am doing a good job as a mother”, participants demonstrated both new-found assurance and re-assurance in their capabilities. Five participants noted increased confidence as the primary message from the program, with Participant 9 noting, “I’m a lot more relaxed and less worried about whether I’m doing the right thing.” Three participants felt more able to help their baby. Another four participants felt more connected, and Participant 21 commented “just the fact that I know he is responding to me, makes me feel I’m increasing my sense of well-being, ‘cause I feel like I am doing a good job”.

Participants were also asked about their experience of the program and if they felt it had influenced their relationship with their baby. The strongest messages from the program content were new realizations about the time it takes for a baby to “process what you’re saying” (13 participants), and the idea that babies can look away when they’re over-stimulated (8 participants). The strongest new realizations about their own capability after the program were that they could sing to soothe or settle their baby (14 participants), and after the program they were more confident in reading cues (6 participants). When asked how Time together had influenced their behavior with their baby, eleven participants reported that they felt more connected to their baby. Participant 10 noted, “I’d say, I connect with her on a deeper level much more often”, arising from an earlier comment that she was now much more aware of the heightened moments of attunement that have been referred to as ‘Moments of Meeting’ [28]. Six participants noted that “It feels like we’re *really* communicating”, and a further four felt more confident in their interpretation of their baby’s efforts to communicate. Three participants did note that they were singing more but they felt this was aligned with their baby’s natural development anyway.

## 4. Discussion

The aim of the present study was to evaluate the impact of *Time Together* on measures of self-efficacy (KPCS), infant bonding (MIBS), depression (EPDS), and anxiety (STAI) in new mothers in a community setting. Originally developed for clinical settings, *Time Together* aims to help parents reflect upon infant and caregiver behaviors through a discussion of how the ways in which they use their voice can create, support and modulate interplay with their baby. The program’s specific objectives were to provide mothers with tools to strategically achieve caregiver goals via the use of the voice.

Significant improvement was seen at the group level for maternal self-efficacy (KPCS), with a large effect size (0.69), which is notable in the context of such a brief psycho-educational program (90-min single session). Indeed, a meta-analysis carried out by Bakermans-Kranenburg and colleagues [29] reported that psycho-educational programs with few sessions produced the most effective outcomes when aimed at parental sensitivity and behavior. The increase in parental confidence is consistent with previous literature showing a particular benefit for promoting maternal-self-efficacy when mother-infant psycho-educational programs are delivered early in the infant’s life [29]. In particular, programs targeted at infants between 0–2 years of age have proven to be effective by providing at-risk parents with educational tools to facilitate sensitive and responsive behavior [30]. Moreover, confidence in parenting has been linked to stimulating caretaking [31], sensitivity, warmth [14] and responsiveness [32], all of which are essential for optimal infant development [33] 

The other three measures did not change, due to floor effects resulting from low levels at baseline of depression and anxiety and an absence of issues relating to mother-infant bonding in this particular community sample. It remains an open question whether the *Time Together* program would yield improvements on these measures in at-risk groups where baseline measures are more likely to be in the clinical or sub-clinical range. 

The deductive content analysis elicited themes which further emphasize the shift in participants’ confidence in reading and responding to their baby’s cues. As a brief program, *Time Together* did stimulate participants’ confidence in both understanding and responding to their baby’s cues. As a fundamental part of parenting confidence, noticing and interpreting their infant’s cues is a lynchpin to sensitive interaction. Participants described increased confidence, feeling less stressed and more connected to their baby. This provides a promising alignment with Spinelli’s findings [15] that interventions focused on parental vocabulary and sensitive responsiveness may serve to enhance parental communicative and caregiving competencies. 

The program provided an acceptable and practical experience from which the participants reported that the strongest messages were a new understanding of their baby’s behaviors, and a new sense of their own capacity to purposefully respond to their baby with their voice. The participants’ statements of confidence in responding, enticing and soothing their baby are indicative perhaps of Spinelli’s notion of empathy [15]. While their awareness of how useful their own vocal, facial and gestural communication could be for their baby is supported by Golinkoff’s finding [32] that attuned infant directed speech fosters communicative skills. 

While this small-scale trial provides promising results, further research is needed to test the effect of the program for a wider caregiver groups, including fathers, and other significant people in the baby’s life. As the program relies on the therapist to tailor the program during implementation, program fidelity trials are needed before larger scale research is warranted. Implications for practice remain more evident for infants who are likely to experience altered opportunities for interaction with attuned adults either through health or social risk in the infant or parent. 

## 5. Conclusions

The contribution of the *Time Together* program is that a one-time 90 min session can provide encouraging results in the key area of maternal self-efficacy. The program makes no attempt to seek out and deconstruct traits but rather remains pivotally focused on the existing capacities of parent, and supporting them to imagine their own do-able actions. This feasibility study demonstrated that a brief voice-centered psycho-educational program that emphasizes the pre-existing communicative capacities of new mothers, provides promising outcomes for improved maternal self-efficacy as a key component of early parenting. 

## Figures and Tables

**Table 1:** Baseline demographics.

**Mother’s Age (Years), Mean ± SD.**		34.8 ± 4.3
**Baby’s Age (Weeks), Mean ± SD**		9.6 ± 2.5
Status	Employed	11 (61%)
	Unemployed	7 (39%)
	Studying	0
Education	No formal qualification	0
	GCSE/A level	2 (11%)
	Undergraduate	9 (50%)
	Postgraduate	7 (39%)
First time parenting		16 (89%)
Attend baby class/es		9 (50%)

**Table 2:** Pre-post comparison for measures of interest.

Scheme	Mean Rank (pre)	Mean Rank (post)	Statistic	*p*-Value
EPDS	8.85	7.92	*Z* = −1.07	>0.05
STAI	6.69	7.50	*Z* = −0.56	>0.05
MIBS	4.67	4	Z = −1.51	>0.05
KPCS	4.33	9.46	Z = −2.8,	<0.004

One-tailed *t*-tests and Wilcoxon signed rank tests for pre-post measure comparisons.

## Data Availability

Data available on request due to restrictions e.g., privacy or ethical. The data presented in this study are available on request from the corresponding author. The data are not publicly available due to privacy.

## Appendix A

Concepts of self-efficacy^23^ (Črnčec, Barnett, and Matthey, 2008)

1. I am confident about feeding my baby
2. I can settle my baby
3. I am confident about helping my baby to establish a good sleep routine
4. I know what to do when my baby cries
5. I understand what my baby is trying to tell me
6. I can soothe my baby when he / she is distressed
7. I am confident about playing with my baby
8. If my baby has a common cold or slight fever, I am confident about handling this
9. I feel sure that my partner will be there for me when I need support
10. I am confident that my baby is doing well
11. I can make decisions about the care of my baby
12. Being a mother / father is very baby stressful for me
13. I feel I am doing a good job as mother / father
14. Other people think I am doing a good job as a mother / father
15. I feel sure that people will be there for me when I need support
